# An Alternative and Efficient Topicalization Technique for the Difficult Airway: A Case Series

**DOI:** 10.7759/cureus.89259

**Published:** 2025-08-02

**Authors:** Rachel H Seet, Jocelyn Ong, Avinash Gobindram, Jessica E Malanjum

**Affiliations:** 1 Anaesthesia and Surgical Intensive Care, Changi General Hospital, Singapore Health Services, Simei, SGP; 2 Anaesthesiology, Singapore General Hospital, Singapore Health Services, Outram, SGP

**Keywords:** airway management, difficult airway, fiberoptic bronchoscopy, intratracheal intubation, nebulizers and vaporizers, oxygen inhalation therapy, topical airway anaesthesia

## Abstract

Effective airway topicalization is essential for awake tracheal fiberoptic intubation (ATI) in patients with a difficult airway. Traditional methods often result in inadequate anesthesia at the laryngeal inlet, leading to patient discomfort, procedural difficulty, and excessive local anesthetic (LA) use.

This case series introduces a novel, resource-efficient topicalization technique using standard operating theatre equipment. It employs two custom assemblies: one targets LA precisely at the laryngeal inlet, while the second augments spray-as-you-go application with supplemental airflow to enhance LA dispersion, oxygenation, and secretion clearance. Currently, no existing device combines all of these functions into a single, integrated system.

In four varied cases, including patients with obstructive airway pathology, cystic hygroma, and oropharyngeal tumors, this method enabled smooth, first-attempt ATI with minimal patient discomfort.

This demonstrates a feasible, effective, and procedurally efficient technique. A custom device (Avi Airway) is under development at our institution to integrate these features into a streamlined system. Further studies are planned to assess its broader clinical utility.

## Introduction

Effective airway topicalization is essential to facilitate a successful and smooth awake intubation in patients presenting with a difficult airway. In the majority of such cases, awake intubation is deemed the gold standard and the most appropriate method for securing the airway, as it allows for continuous spontaneous ventilation and maintenance of airway patency [1,2]. First-attempt success is imperative, as each subsequent attempt is associated with adverse consequences such as airway trauma, obstruction, and bleeding [1]. Traditionally, effective topicalization of the airway involves a combination of gargles, sprays, nebulizers, atomizers, and/or airway blocks [3]. However, many of these techniques may result in ineffective delivery of local anesthesia (LA) [4], and are unable to directly guide the LA to the laryngeal inlet, leading to patient discomfort and coughing as the scope passes through the vocal cords. This can potentially result in higher doses of LA being used or failure to achieve first-attempt success. In addition, the working channel of the flexible bronchoscope is narrow, and clearance of secretions, especially if viscous, is difficult [5,6]. The spray-as-you-go technique (where LA is delivered intermittently through the bronchoscope’s working channel during advancement) allows for direct visualization while administering LA, but its reliability is dependent on both the patient and operator for successful deposition at the desired site. Transtracheal deposition of LA (where LA is injected percutaneously through the cricothyroid membrane to anesthetize the trachea via reflex coughing) provides reliable delivery to the laryngeal inlet but is invasive, requires accurate localization of the cricothyroid membrane, and carries potential complications.

This case series explores and presents an alternative technique for airway topicalization, utilizing equipment commonly found in the operating theatre. These methods aim, firstly, to improve the efficiency of LA delivery to the laryngeal inlet and vocal cords; secondly, to offer an alternative to suctioning for improved secretion management; and thirdly, to demonstrate the feasibility of achieving the above using readily available operating theatre equipment. Based on the conceptual feasibility and efficiency of these methods, we also plan to develop a simple prototype (Avi Airway) that integrates the features and benefits of all these techniques. This device aims to provide more efficient topicalization for awake tracheal intubation.

## Case presentation

Description of an alternative technique for airway topicalization

This alternative technique is centered around two equipment assemblies.

**Figure 1:**
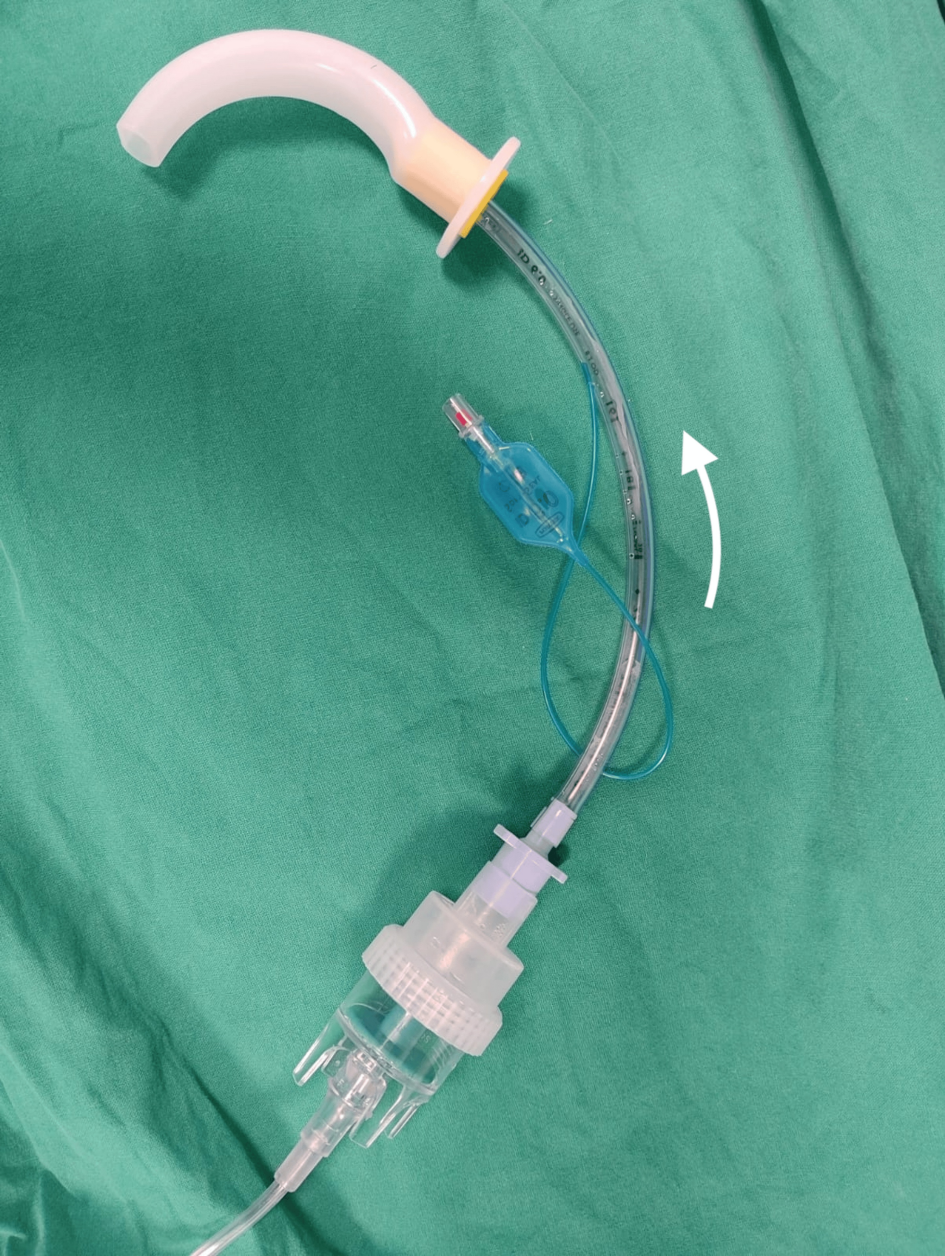
The first equipment assembly. The white arrow indicates the direction of oxygen flow.

The first assembly is demonstrated in Figure 1.

**Figure 2:**
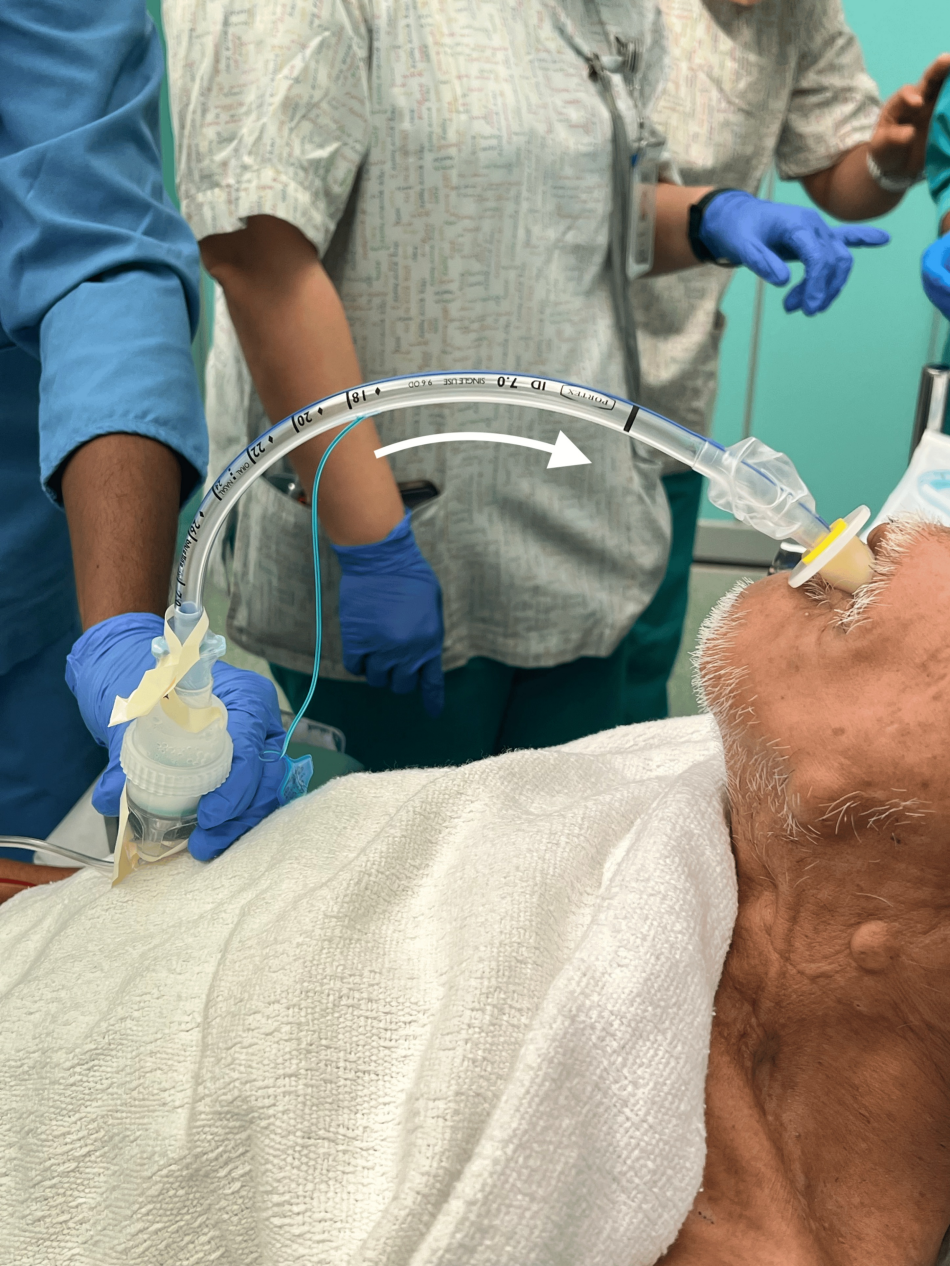
Placement and usage of the first equipment assembly. The white arrow indicates the direction of oxygen flow. This patient's clinical details are described in Case 1.

This is an assembly of connected equipment in the following order: oxygen tubing (connected to an auxiliary oxygen outlet running at 8 L/min), nebulizer chamber (containing 4 ml of 2% lignocaine), endotracheal tube (ETT), Guedel airway. If possible, we suggest using the ETT of choice for subsequent intubation to reduce wastage; otherwise, a size 6 ETT fits well with this assembly. The Guedel airway is then inserted by the patient into their own mouth and advanced as tolerated, while being gently held in place by the anesthetic team during nebulization (Figure 2). Sizing of the Guedel airway is in accordance with standard sizing, from the corner of the patient’s mouth to the angle of the mandible [7].

Before this is done, the posterior tongue to the oropharynx needs to be topicalized either with LA spray or directly via nebulization using this equipment assembly. The former was used in Case 1 and the latter in Case 4. This technique aims to direct the LA to specifically target the laryngeal inlet. It is necessary to interpose an ETT between the oral airway and the nebulizer to keep the nebulizer chamber upright. The length of the ETT is retained for two reasons: firstly, to provide the curvature required for the nebulizer chamber to remain upright, if the ETT were shorter, the chamber may be slanted, resulting in inefficient nebulization. Secondly, we try to use the same ETT for subsequent intubation where possible, saving an additional piece of equipment.

**Figure 3:**
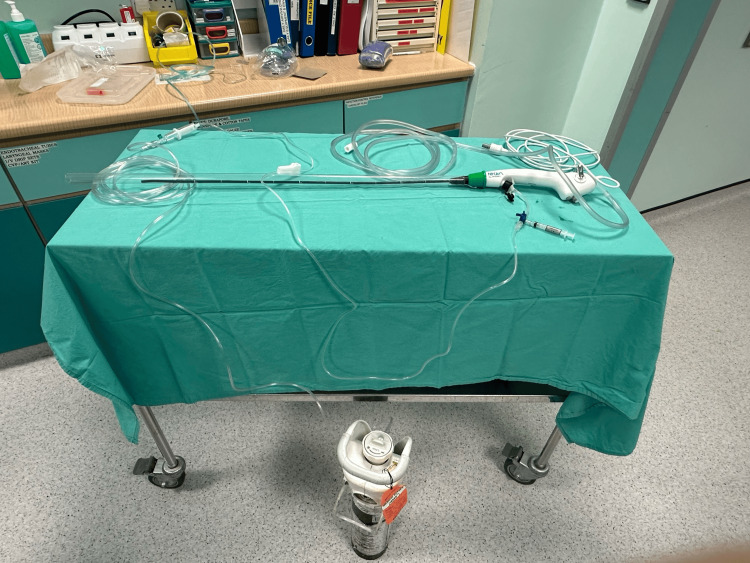
The second equipment assembly.

**Figure 4:**
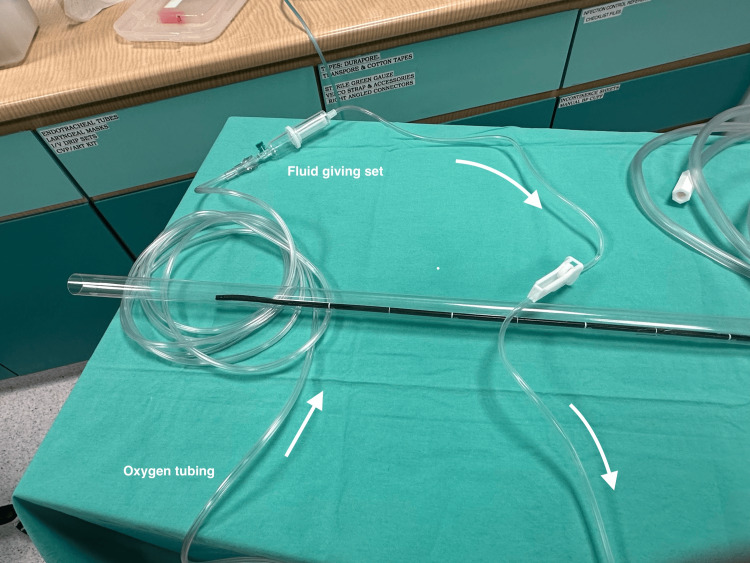
First close-up view of the second equipment assembly. The white arrows indicate the direction of oxygen flow.

**Figure 5:**
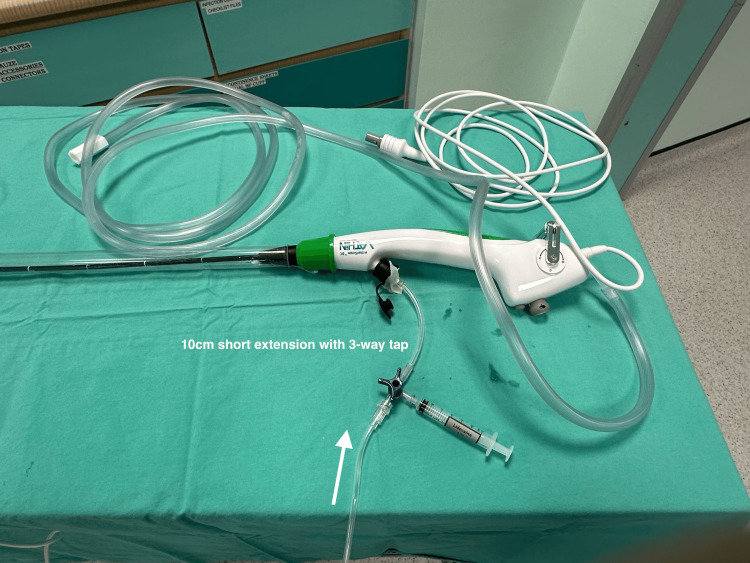
Second close-up view of the second equipment assembly. The white arrow indicates the direction of oxygen flow.

The second assembly is demonstrated in Figure 3. This assembly of equipment follows the order of: oxygen tubing (connected to a standalone oxygen cylinder), fluid giving set, 10 cm short extension with a 3-way tap, working channel of the Ambu fiberoptic bronchoscope (Figures 4-5 for a close-up view).

During navigation of the bronchoscope, oxygen at a flow rate of 2-4 L/min is maintained via the working channel to aid in the clearance of secretions as an alternative to suctioning. Only once the vocal cords are visualized, a final spray of 4% lignocaine (2-3 ml) can be given via the 3-way tap with the oxygen flow rate at 10 L/min, prior to advancing the ETT. This enhances the traditional spray-as-you-go technique with additional airflow that helps to disperse LA, increase oxygen delivery, and improve secretion management. Oxygen flow via the working channel is ceased immediately after placement of the ETT while the bronchoscope is removed.

These equipment assemblies have facilitated smooth, single-attempt intubations in the following four cases. As the primary objective of this case series is to describe the airway topicalization technique, sedation practices were not standardized. Anaesthetic providers were permitted to use their preferred sedation regimens, and these have been reported accordingly for each case to reflect real-world variability.

Case 1

A 70-year-old man with a background of GOLD E chronic obstructive pulmonary disease (COPD) on long-term oxygen therapy (1-2 L/min) and bronchiectasis presented with a painless base of tongue swelling over a few weeks. He was planned as a semi-elective case for awake fiberoptic intubation (AFOI), tracheostomy, panendoscopy, base of tongue tumor biopsy, and nasogastric tube (NGT) insertion.

On the day of surgery, the patient’s baseline oxygen saturation was 88-89% on room air, with a baseline wheeze. He had a Mallampati score of 4.

**Figure 6:**
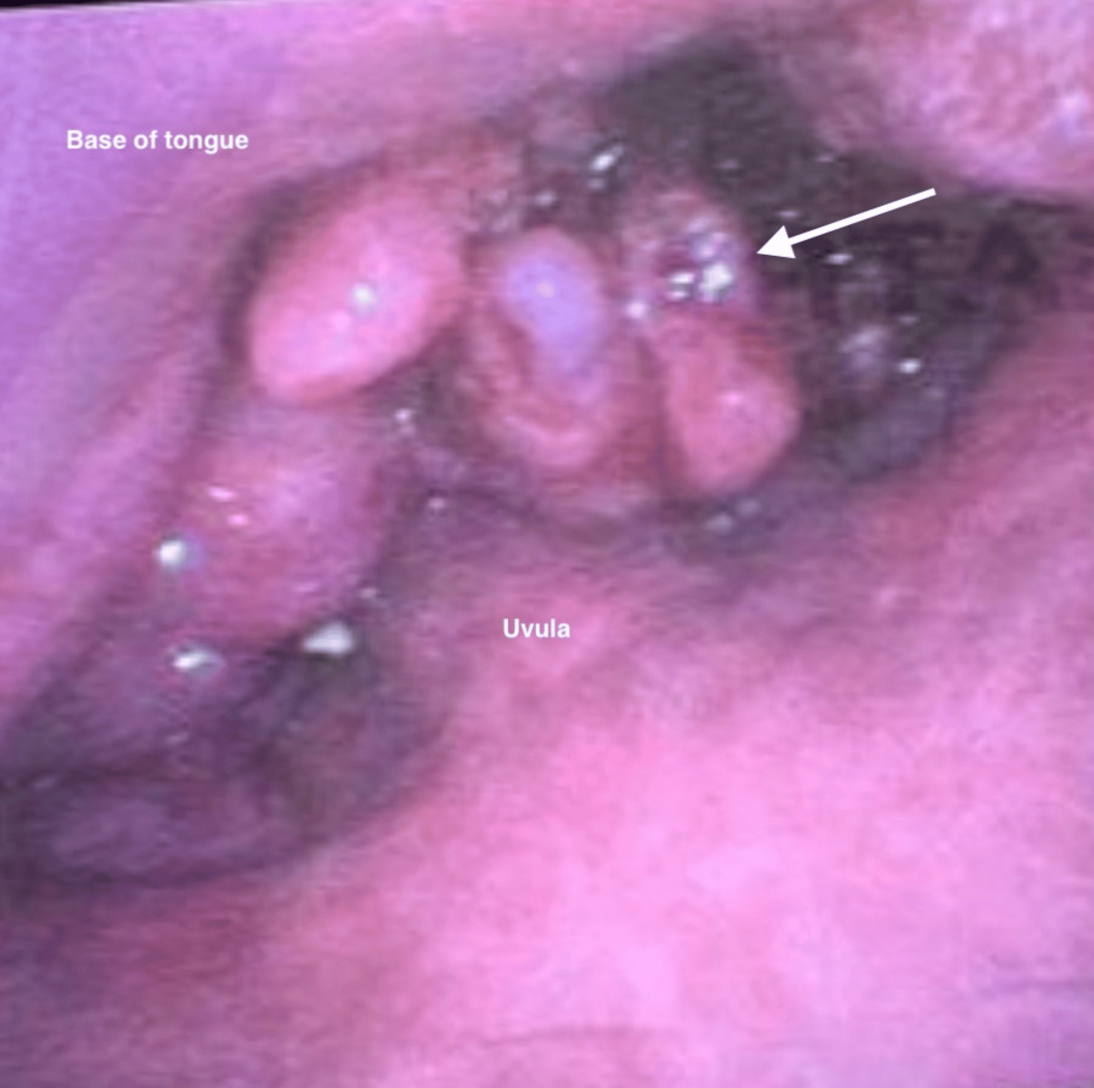
Case 1: Fiberoptic view of tongue base tumor (denoted by white arrow).

Fiberoptic bronchoscopy revealed a large exophytic tongue base tumor encompassing the entire posterior one-third of the tongue (Figure 6).

Airway topicalization was performed using the above-described approach (as previously depicted in Figure 2), resulting in smooth awake oral intubation with a size 7 ETT on the first attempt. Total lignocaine dose used for topicalization was 6 mg/kg.

High-flow nasal cannula settings of 90% FiO₂ at 50 L/min were used during airway management in view of the patient’s poor baseline saturation. SpO₂ remained at 100%, with no significant hemodynamic changes throughout ATI.

Sedation was provided using TCI remifentanil at 1.0 ng/ml (effect site).

Case 2

**Figure 7:**
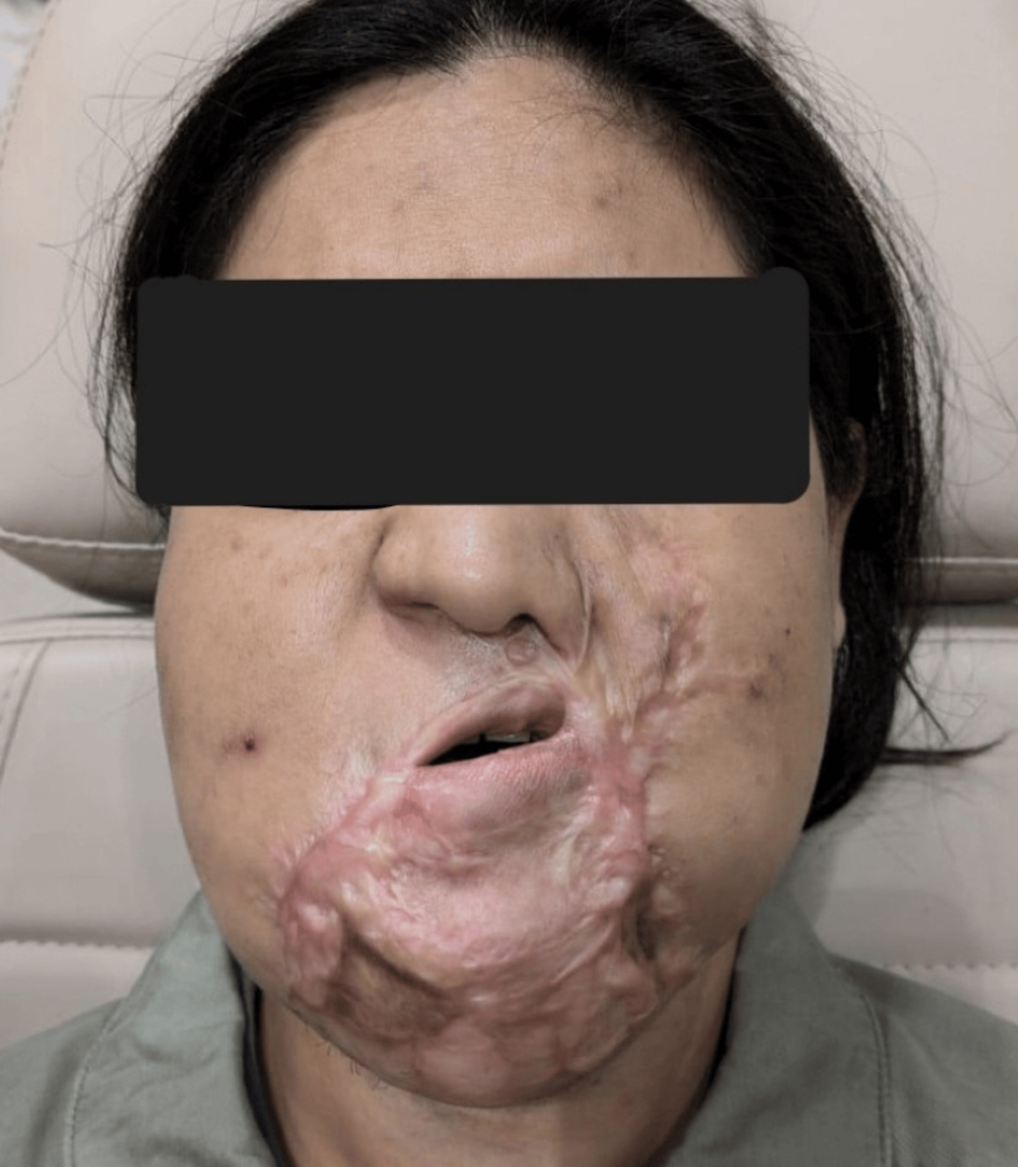
Case 2: Demonstration of maximum mouth opening and significant scar tissue resulting in a difficult airway.

A 27-year-old female with a history of cystic hygroma and multiple previous surgical interventions was electively admitted for release of left commissural cicatricial contracture and free radial forearm flap reconstruction of the left lip. Her prior surgical history included repeated excisions at 3 months, 5 months, 4 years, 10 years of age, and sclerotherapy at 25 years. Her maximum mouth opening was one finger’s breadth, with significant scar tissue (Figure 7), and a Mallampati score of 4. A nasal intubation was planned.

**Figure 8:**
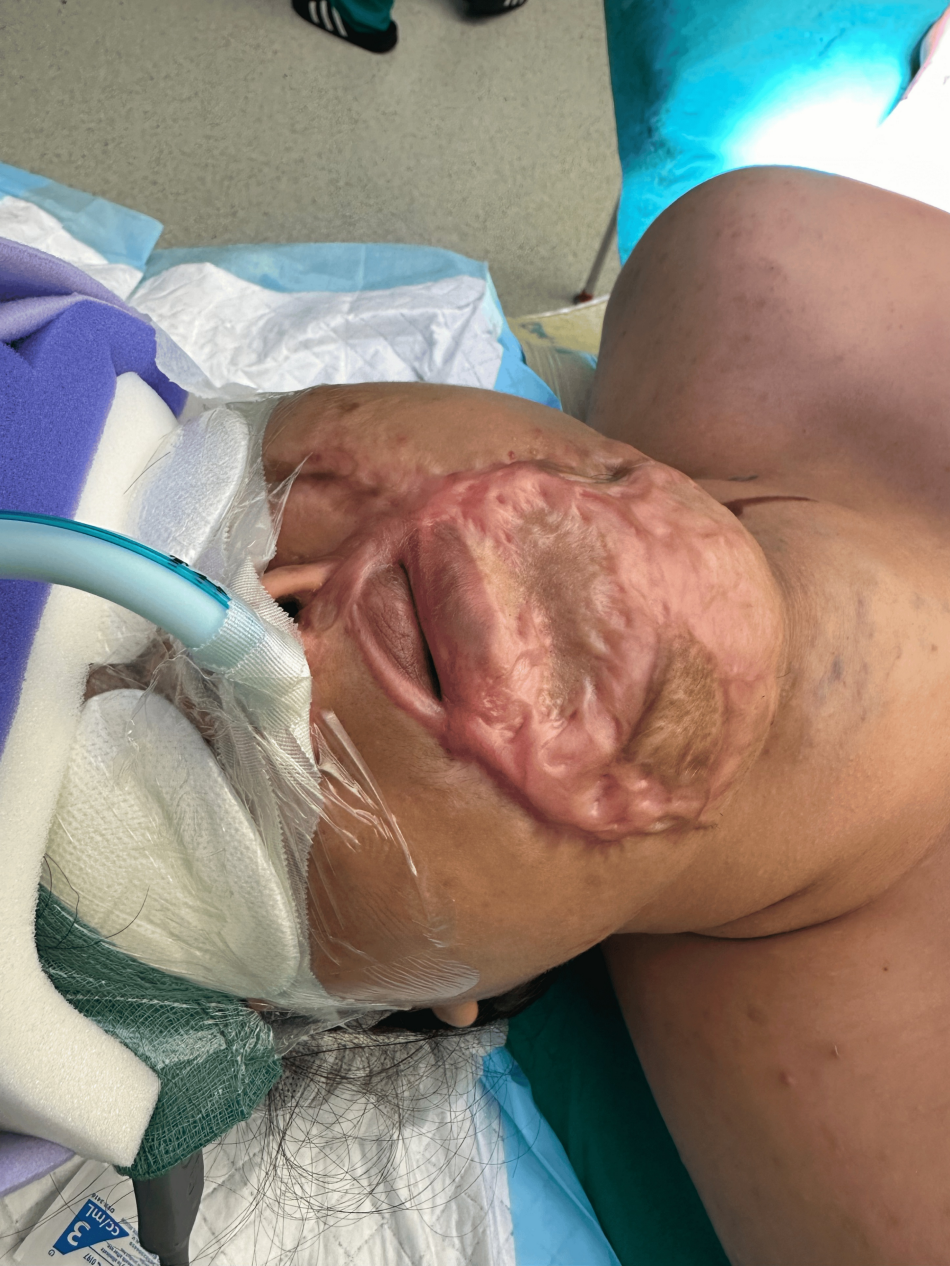
Case 2: Post-intubation view.

Airway topicalization was performed using the above approach, with co-phenylcaine used to anesthetize the nasal mucosa. This resulted in smooth awake intubation with a size 6.5 North RAE Ivory ETT via the right nostril on the first attempt (Figure 8). Total lignocaine dose used for topicalization was 5.5 mg/kg. SpO₂ remained at 100%, with no significant hemodynamic changes throughout ATI. The patient expressed satisfaction with the intubation process during review the following day.

Sedation was provided using TCI remifentanil at 0.9 ng/ml (effect site) and dexmedetomidine at 0.5 mcg/kg/h.

Case 3

**Figure 9:**
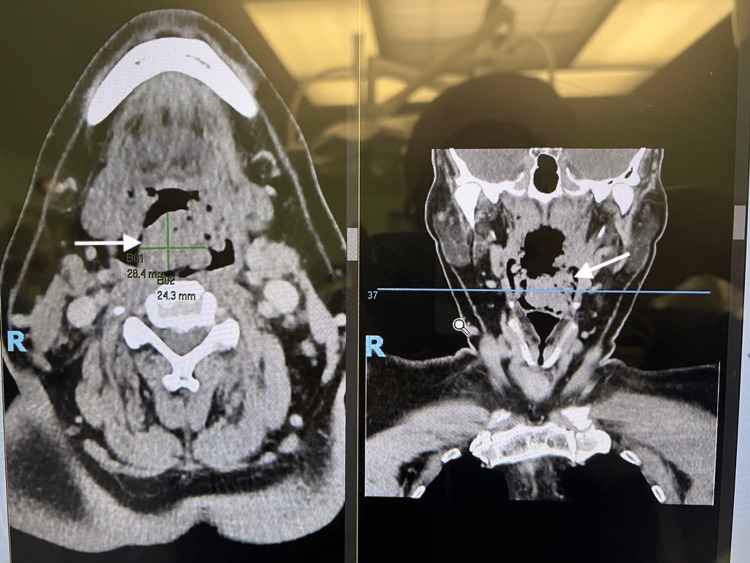
Case 3: CT neck showing adenoidal hyperplasia with enlargement of the central nasopharyngeal soft tissues and bilateral palatine tonsils (denoted by white arrows).

A 50-year-old man with a background of severe obstructive sleep apnea (Apnea-Hypopnea Index 70), obesity hypoventilation syndrome, obesity (BMI 38), and an epiglottic mass was electively admitted for panendoscopy, biopsy of the epiglottic mass, bilateral tonsillectomy, and lingual tonsil excision. Preoperative CT of the neck showed features suggestive of adenoidal hyperplasia with enlargement of the central nasopharyngeal soft tissues and bilateral palatine tonsils. There was also a nodular soft tissue density lesion inseparable from the epiglottis, of similar density but indeterminate nature (Figure 9). He had a Mallampati score of 3.

Airway topicalization was performed using the above approach, with co-phenylcaine to topicalize the nasal mucosa. This resulted in a smooth awake intubation with a size 7 armored ETT via the left nostril on the first attempt. Total lignocaine dose used for topicalization was 4.9 mg/kg. SpO₂ remained above 95%, and there were no significant hemodynamic changes throughout ATI. The patient expressed satisfaction with the intubation process during a review the next day.

Sedation was provided with TCI remifentanil running at 0.5-1.0 ng/ml (effect site).

Case 4

**Figure 10:**
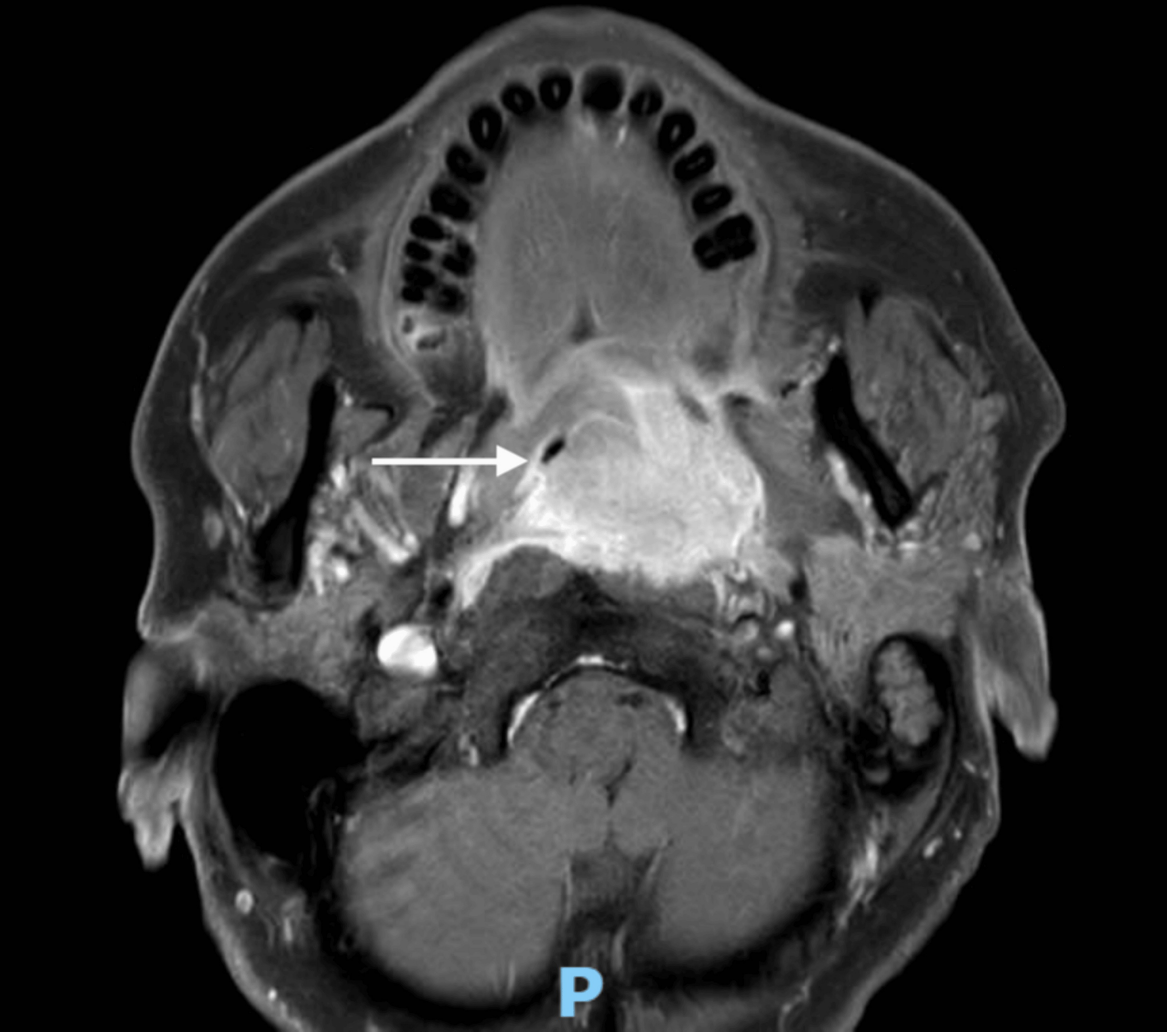
Case 4: MRI neck showing a heterogeneous, enhancing lobulated mass (denoted by white arrow) with lobulated margins, approximately 4.7 cm × 4.2 cm × 3.0 cm, centered at the left tonsillar fossa, with local extension to the tongue base/soft palate, nasopharynx, retropharyngeal/prevertebral, and parapharyngeal spaces.

A 61-year-old man with a left tonsillar tumor extending up to the post-nasal space was electively admitted for biopsy, rigid esophagoscopy, and examination under anesthesia. Preoperative MRI of the neck showed a heterogeneous, enhancing, lobulated mass with lobulated margins, approximately 4.7 cm × 4.2 cm × 3.0 cm, centered at the left tonsillar fossa with local extension to the tongue base, soft palate, nasopharynx, retropharyngeal/prevertebral, and parapharyngeal spaces. It crossed the midline, resulting in near-complete effacement of the naso/oropharynx (Figure 10). He had a Mallampati score of 4.

Airway topicalization was performed using the above approach, which resulted in smooth awake oral intubation with a size 6 ETT on the first attempt. Total lignocaine dose used for topicalization was 2.2 mg/kg. SpO₂ remained at 100%, and there were no significant hemodynamic changes throughout ATI.

Sedation was provided with TCI remifentanil running at 0.6 ng/ml (effect site) and TCI propofol running at 0.6 mcg/ml (effect site).

## Discussion

A well-topicalized airway ensures patient comfort and procedural success during ATI. The upper airway is usually easily topicalized using traditional techniques for LA delivery, such as nebulization, atomization, and LA sprays. However, suboptimal anesthetic coverage of the laryngeal inlet increases patient discomfort and procedural challenges, where patients tend to cough or gag when the flexible bronchoscope or ETT is passed through the vocal cords. Our proposed two-stage technique allows nebulization through the Guedel to the upper airway and laryngeal inlet with the first assembly of equipment, and the second assembly facilitates targeted, visualized LA spray to the glottis and vocal cords via the working channel of the flexible bronchoscope, with enhanced secretion management.

This case series demonstrates the feasibility and effectiveness of our proposed technique while utilizing readily available operating theatre equipment.

The key advantages of this approach include:

Targeted LA delivery

Our method for airway topicalization aims for precise deposition of LA to the laryngeal inlet, a region often inadequately anesthetized with conventional methods. By targeting this area more effectively, we observed reduced coughing, a challenge also noted by Alfieri A et al. [8], as well as improved patient tolerance during intubation. This may facilitate procedural efficiency, reflected in smooth first-attempt successes in ATI across all four cases in this series.

Secretion management and oxygen insufflation

Oxygen at a controlled flow rate effectively displaces secretions and serves as a viable alternative to suctioning, which is often ineffective during AFOI. This was previously observed by Rajan S et al. [9] in a prospective randomized study comparing suction versus oxygen insufflation during AFOI, and was also reviewed by Garioud A and Kristensen MS [10]. In the review, oxygen flow rates between 2-7 L/min were described; in this case series, we opted for the conservative end of the range, using oxygen flows between 2 and 4 L/min. This feature is particularly beneficial in patients with excessive secretions or compromised respiratory function, as demonstrated in Case 1 (COPD and bronchiectasis) and Case 4 (tonsillar tumor with significant airway compromise). Furthermore, it provides oxygen supplementation throughout the process of AFOI.

Minimization of LA requirements

More efficient LA delivery may allow for lower total doses, reducing the risk of systemic toxicity. As highlighted by Kostyk P et al. [3], the known limit of 7-9 mg/kg of lignocaine dosage for topicalized LA should be avoided in routine clinical practice due to variable pharmacokinetics and patient factors.

Utilization of existing equipment

By repurposing commonly available equipment, these methods provide a practical solution when specialized topicalization devices are unavailable.

Despite these advantages, several limitations exist. First, the technique currently requires multiple steps prior to intubation, which can be time-consuming. This may pose challenges in urgent or time-sensitive airway management scenarios. Additionally, while the first assembly can theoretically be used alone to topicalize the entire posterior tongue and oropharynx (as in Case 4), this process requires patience and patient cooperation and may not always be feasible in emergent situations. Thirdly, with oxygen being delivered via the suction port of the fiberoptic scope, care must be taken to ensure the tip of the scope does not inadvertently breach soft tissue or mucosa, resulting in surgical emphysema, or enter the esophagus, which could cause gastric distension and potentially catastrophic events such as gastric rupture and pneumothorax [10]. Oxygen delivery should therefore be ceased if there is any doubt about the positioning of the scope’s tip. Finally, as this was a retrospective case series, standardized patient-reported comfort scores were not collected, which limits our ability to objectively quantify the patient experience.

Recognizing these limitations, we are developing a novel device, the Avi Airway, which integrates these techniques into a single, streamlined system. This device aims to simplify the process by enabling targeted nebulization and oral intubation through the same piece of equipment, with easy removal post-intubation. The conceptual feasibility of this device is demonstrated by the success of the techniques described in this series. Future studies will focus on evaluating its efficacy in a larger patient cohort for widespread clinical use.

Our findings align with previous literature on optimizing airway topicalization and management during AFOI. Prior studies have explored various approaches, including high-flow nasal oxygen to maintain airway patency, enhance patient comfort, and improve mucociliary clearance during AFOI [11], various LA delivery methods [12], as well as strategies to mitigate coughing during intubation [8]. However, this case series describes an alternative approach that integrates the considerations of targeted LA delivery, secretion management, and optimizing patient comfort, using pre-existing operating theatre equipment.

## Conclusions

In conclusion, our alternative method for airway topicalization offers a practical, efficient, and targeted approach to managing the difficult airway. It leverages existing equipment to address the limitations of traditional techniques, while prioritizing patient safety and comfort. The future development of the Avi Airway represents a promising next step in refining this process, with the potential to further enhance the ease and efficacy of awake intubation in challenging cases.
